# Supplementary material for: No Evidence that Predictions and Attention Modulate the First Feedforward Sweep of Cortical Information Processing
Source: Cereb Cortex. 2019 Mar 16;29(5):2261–78. doi: 10.1093/cercor/bhz038 (PMC6484894; doi:10.1093/cercor/bhz038)
Supplement: Supplementary Data [file bhz038_supplementary_materials.zip › bhz038_Alilovic_et_al_supplementary_legends.pdf]

Alilovic et al supplementary legends

Figure S1. Individual subject scalp topographies and ERPs from the cueing task in Experiment 1. This figure shows for each subject separately, C1 scalp distributions at 80ms post-stimulus for stimuli presented in the upper and lower visual field (first two columns) and P1 scalp topographies at 100ms for lower visual field stimuli (third column). The C1 peak channels are marked in black. The fourth and fifth column of the figure show individual ERP waveforms to attended (A) and unattended (UA) upper and lower stimuli, and predicted (P), non-predicted (NP) and unpredicted (UP) upper and lower stimuli, respectively.

Figure S2. Individual subject scalp topographies and ERPs from the cueing task in Experiment 2.

This figure shows for each subject separately, C1 scalp distributions at 80ms post-stimulus for stimuli presented in the left and right upper visual field (first two columns) and P1 scalp topographies at 100ms for right upper visual field stimuli (third column). The C1 peak channels are marked in black. The fourth and fifth column of the figure show individual ERP waveforms to attended (A) and unattended (UA) left and right upper field stimuli, and predicted (P), non-predicted (NP) and unpredicted (UP) left and right upper field stimuli, respectively.
